# EFFECTS OF MINDFUL FELDENKRAIS EXERCISES AND STRENGTH TRAINING ON COGNITIVE EXECUTIVE FUNCTION IN OLDER ADULTS

**DOI:** 10.1093/geroni/igz038.2410

**Published:** 2019-11-08

**Authors:** Gerhild Ullmann, Yuhua Li, Meredith A Ray

**Affiliations:** 1 University of Memphis, School of Public Health, Memphis, Tennessee, United States; 2 University of Memphis - School of Public Health, Memphis, Tennessee, United States

## Abstract

Decline in cognitive function associated with aging is one of the greatest concerns of older adults and often leads to significant burden for individuals, families, and the health care system. This 3-arm randomized controlled trial (RCT) responds to the urgent need to identify strategies which can enhance and/or maintain cognitive vitality in older adults. The study is funded by the National Institute on Aging, and aims to examine the effects of both the mind-body exercise Feldenkrais and strength training on cognitive executive function in independent living older adults (N=90) age 65 to 85. Participants of the first wave (n=45) were randomized to a (1) Feldenkrais group, (2) strength training and (3) no-intervention control group. Intervention groups met twice a week for 12 weeks. Cognitive and physical performance measures of the NIH-Toolbox were used at baseline, post-intervention and at a 3-month follow-up. Results of changes in cognitive executive functions within and across groups will be presented. The findings will suggest if such interventions would be a viable low-cost option for older adults to maintain cognitive vitality and thereby impact the development of programs and guidelines for combatting decline in cognitive function.

